# The Role of Annual Flowering Plant Strips on a Melon Crop in Central Spain. Influence on Pollinators and Crop

**DOI:** 10.3390/insects11010066

**Published:** 2020-01-20

**Authors:** Celeste Azpiazu, Pilar Medina, Ángeles Adán, Ismael Sánchez-Ramos, Pedro del Estal, Alberto Fereres, Elisa Viñuela

**Affiliations:** 1 Escuela Técnica Superior de Ingeniería Agronómica, Alimentaria y de Biosistemas, Universidad Politécnica de Madrid (ETSIAAB-UPM), Avenida Puerta de Hierro 2-4, 28040 Madrid, Spain; pilar.medina@upm.es (P.M.); angeles.adan@upm.es (Á.A.); pedro.delestal@upm.es (P.d.E.); elisa.vinuela@upm.es (E.V.); 2 Unidad Asociada IVAS (UPM-CSIC): Control de Insectos Vectores bajo sistemas de Agricultura Sostenible, 28040 Madrid, Spain; a.fereres@csic.es; 3 Instituto Nacional de Investigación y Tecnología Agraria y Alimentaria, O.A., M.P. (INIA), Carretera de la Coruña Km 7.5, 28040 Madrid, Spain; ismael@inia.es; 4 Instituto de Ciencias Agrarias, Consejo Superior de Investigaciones Científicas (ICA-CSIC), Serrano 115 Dpdo, 28006 Madrid, Spain

**Keywords:** agri-environment scheme, ecosystem services, wild bees, attractiveness, interspecific competition, facilitation

## Abstract

Planting flower strips adjacent to crops is among the habitat-management practices employed to offer alternative floral resources to pollinators. However, more information is needed to understand their potential spill-over of pollinators on nearby insect-pollinated crops. Over the course of two consecutive years, the suitability of a flower mixture of 10 herbaceous plants for pollinators was evaluated on a weekly basis, in a randomized block design of two melon plots (10 × 10 m^2^) with or without 1 m-wide flower strips. Floral coverage and pollinator visits to the plant species, as well as pollinator visits and the yield and quality of the crop, were assessed. Additionally, the selected mixture was tested for 1 year in a commercial field in order to ascertain how far the flower strip could influence visitors in the crop. The most suitable species for a flower strip in central Spain based on their attractiveness, floral coverage and staggered blossom were *Coriandrum sativum* L., *Diplotaxis virgata* L., *Borago officinalis* L. and *Calendula officinalis* L. The flower strip can act as either pollinator competitor or facilitator to the crop, depending on their floral coverage and/or the predominant species during the crop bloom period. The concurrence of blooming of the rewarding plant *C. officinalis* with the melon crop should be avoided in our area. In the commercial field, the bee visitation rate in the melon flowers decreased with the distance to the flower strip. No influence of the specific flower strip evaluated on crop productivity or quality was found.

## 1. Introduction

Insect pollinators are essential in both natural and agricultural ecosystems due to their role in plant reproduction [1,2,3]. The European honeybee (*Apis mellifera* L.; Hymenoptera: Apidae)—the most economically valuable and managed pollinator worldwide—has been reported to visit the greatest variety of crop species in the world [1], and its hives are regularly introduced in many pollinator-dependent crops. In recent decades, there have been many reports of unusually high rates of honeybee colony losses from many parts of the world, sometimes attributed to colony collapse disorder syndrome [4,5,6]. This fact, combined with the increase in agricultural areas that require insect pollination [7], has led to growing worldwide concern regarding the overreliance on a single species to achieve the satisfactory pollination of agricultural crops [8,9]. The role of wild bees and other managed bees is under consideration, because they can often provide equal, superior or complementary service levels compared to those of managed honeybees [10].

However, in intensive agricultural areas, the use of pesticides [11] and the degradation of natural habitats with the subsequent decrease in plant diversity reduces the abundance and richness of pollinators, due to decreases in food availability and nesting opportunities [5,12,13] which, in turn, may result in crop yield deficits [9,14,15].

In the European Union (EU), one of the key aspects of the biodiversity strategy for 2020 is to ensure no net loss of biodiversity and ecosystem services [16]. As such, in recent years, EU agri-environment schemes have encouraged farmers to dedicate 5% of arable land to ecologically beneficial elements, in order to boost the presence of pollinators in commercial farms [17]. A very popular approach in agroecosystems to increase biodiversity is the creation of nectar- and pollen-rich habitats adjacent to the crops [15,18], and their presence in Spain allows the farmers to receive direct EU green payments [19]. Flower strips can contribute to satisfying the needs of wild pollinators. They can provide continuous resources to pollinators beyond the crop bloom interval, covering the flight period of many pollinator species [15], especially in intensive agricultural areas [20]. Therefore, they can improve wild bee reproduction rates [21], and contribute to enhancing their abundance [22], species richness [23], and population persistence [24].

One of the great challenges of designing flower-rich areas is the selection of plants for the mix. The identification of appropriate plants often depends on the region [25]. Choosing native plants ensures a good adaptation to local soil and climatic conditions, a good interaction with local pollinators, and a lesser risk of becoming a weed and competing with the crop [18,26]. In order to attract many diverse pollinators, the candidate flowering plants should exhibit different phenology and morphology [27], because the access to nectar and pollen resources depends—among other factors—on the pollinator flight period, and tongue length and body size, respectively [2,28]. The differences in the attractiveness of individual flower species to pollinators and other beneficial insects has been extensively investigated based on the frequency of visits and/or the flowering duration [29,30,31,32,33], and even computational tools are being applied to help select an optimal mix [34]. A given plant species, when sown in mono-specific strips, can exhibit a higher floral coverage than when sown in mixed flower strips, due to the competition for the space [32]. Therefore, the pollinator visits to this species might be reduced. However, mixed flower strips can support a greater pollinator diversity [35].

Although it is well-known that flower strips support higher insect abundance and diversity than crops [36,37], their potential spill-over of pollinators on nearby insect-pollinated crops is less recognized. Flower plant strips can have a dual role as pollinator competitors or facilitators [38,39,40], and the different plant species play different roles. Whilst some of them can compete for pollinators with the crops, many others do not have a negative effect on the neighboring crop plants and may even play a positive role, when the presence of one plant in bloom increases the presence of pollinators in a nearby plant [40]. In previous studies, when flower strips exported pollinators, the pollination services were found to be increased in some nearby crops (e.g., in mango [38]; tomato [41]; or blueberry [42]), but a lack of effect in many other crops (e.g., cider apple orchards [43], cucumber [44] and summer vegetable crops, [45]), or an effect related to the crop scale (e.g., in strawberries there is positive effect at small plots [46], and a negative effect in commercial plantations [47]) has also been reported. However, when flower strips offer enough pollen and/or nectar, they can concentrate pollinators—thus having a negative effect on the crop [37], and this is a major concern of many farmers [48].

In Central and South Spain, melon (*Cucumis melo* L.) is an economically important summer crop, and its production outdoors and in greenhouses was valued at EUR 217 million in 2017 [49]. Melon plants are andromonoecious, with both male staminate and hermaphrodite flowers producing pollen and nectar; therefore, melon requires bee pollination to improve fruit quality and quantity [50,51], and farmers usually introduce 2–5 *A. mellifera* hives/ha. This crop is also visited around the world by a wide variety of other pollinators, mainly wild bee species [1,45,51,52], harbouring the Halictidae genus *Lasioglossum* in Central Spain, many important key pollinators [52].

In this work, our initial aim was to optimize a flower mix for Central Spain based on the attractiveness, flower coverage and staggered blossom. Secondly, we examined the contribution of the flower mixture to the flower visits in a pollinator-dependent crop (melon), trying to ascertain its role as pollinator competitor or facilitator using experimental plots. Thirdly, the selected mix was tested in a commercial melon field, in an attempt to ascertain up to which distance to the flower strip an effect on visits was detectable in the crop. Finally, we also studied the possible influence of the flower strip on the productivity and quality of the melon crop.

## 2. Materials and Methods

### 2.1. Study Site

The study was carried out in two different areas of Central Spain, both with continental Mediterranean climates (cold winters, hot summers and scant rainfall (≈400–450 mm per year)): in small plots at the experimental farm La Poveda (Arganda del Rey, Madrid; 40°19′ N and 3°29′ W, elevation 536 m above sea level (a.s.l.)), and in a commercial field located in the productive Spanish area of Corral de Almaguer (Toledo; 39°45’ N and 3°11’ W, elevation 708 m a.s.l). The meteorological data, precipitation and temperatures (daily mean, maximum mean and minimum mean temperature), were obtained from the nearest weather stations (1 km from the plots) and are available online at the Agro-climatic Information System for Irrigation [53].

### 2.2. Experimental Design

**Figure 1:**
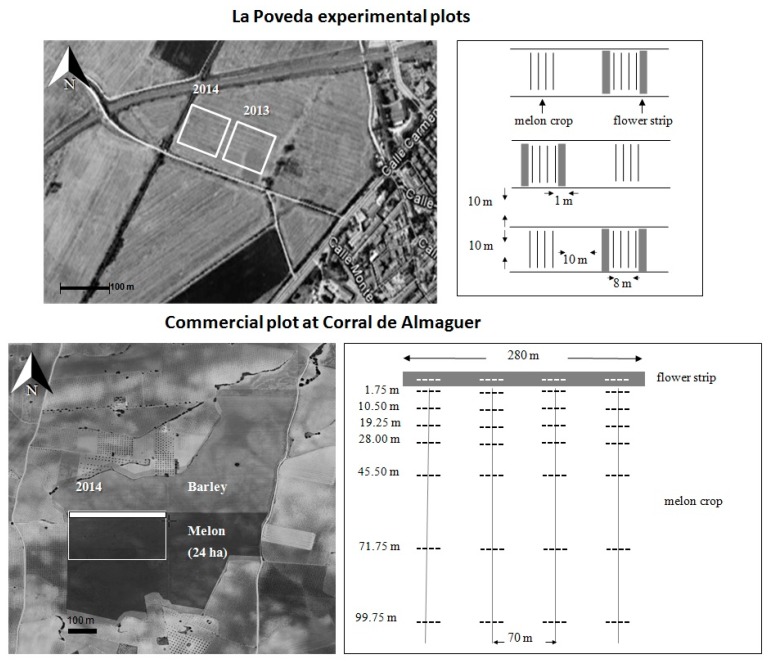
Experimental design and location of La Poveda experimental plots (Madrid; Central Spain) and commercial melon field at Corral de Almaguer (Toledo; Central Spain) with the sampling area (2.8 ha, continuous line).

A 2-year study (2013–2014) was set up in small experimental plots (10 × 10 m^2^) at La Poveda (Madrid). It consisted of a randomized block design of 3 blocks 10 m apart, west–east orientated, with 2 drip-irrigated melon plots 10 m apart each (control and flower plots). The location within the farm was changed every year, depending on the soil availability and irrigation facilities. The spontaneous vegetation in the margins of the control plots was weeded periodically. Flower strips 1 m wide were placed on the two north–south sides of the plots (Figure 1).

A one-year study (2014) was carried out in a commercial field of 24 ha at Corral de Almaguer, where a flower strip (2 m wide × 280 m long) was established in an area of approx. 2.8 ha (100 × 280 m^2^). The experimental design consisted of 4 blocks 70 m apart and with 7 different distances from the flower strip each—1.75, 10.5, 19.25, 28, 45.50, 71.75, and 99.75 m—based on the melon-planting pattern (1.75 m × 1.50 m) and the possible influence of the flower strip (a highest concentration of distances near to the flower strip) (Figure 1).

### 2.3. Melon Crop

Sancho—a hybrid melon cultivar (toad skin type) widely planted in Central Spain—was selected for the study and managed following typical crop cultivation techniques. Pesticide treatments were not applied, except localized, anti-powdery mildew treatments of Flint^®^ (50% trifloxystrobin, water-dispersible granules, 0.25 L/ha, Bayer) up to 3 days before harvesting. Fruits were ready for harvesting at the end of July.

In the experimental plots, melon plants with 2–3 mature leaves were transplanted from mid-May (planted in rows 2 m apart and 1 m between plants). The commercial field was planted on 16 May (planting pattern 1.75 m × 1.50 m). Around the commercial melon field, honeybee hives were routinely managed in order to increase the pollination of this and other adjacent melon fields.

### 2.4. Flower Margin Composition and Growth

We used seed mixes of flowering annual herbaceous plants of different families based on previous work [54,55] (Table 1). All the species were commercially available (Semillas Silvestres SL, Cordoba Spain), and with a seed size suitable for mechanical sowing after adaptation of the machinery—two of the bottle necks when selecting plants for flower mixes. The plant species were native or naturalized, well-adapted to the climate in Central Spain, and had different phenology and flower morphology features (colour, size, corolla shape and depth) [56] in order to attract pollinator diversity. The plants also differed in the floral nectary position, which determines the nectar availability: ‘concealed’ (in deep corollas or spurs), ‘partly-concealed’ (in short corollas) or ‘open’ (in short corollas or in extra-floral nectaries) [43]. For every individual species, we evaluated the attractiveness to visitors, flower coverage, blooming duration and phenology. Based on these parameters, in the experimental farm the composition of the mix was slightly modified in the second year. In the commercial field of Corral de Almaguer, a flower seed mix under commercial development and slightly different to that used in the experimental plots was sown.

Prior to sowing, the soil was prepared by tilling. The seed mix was broadcast-sown and the seeds covered using a shallow stubble plough. In the experimental plots, the sowing was done before winter began (21 December), aiming at reaching 13 plants per m^2^ approx. (optimal density in order to allow every plant to have enough space, 25 × 30 cm^2^). In the commercial field however, the sowing was done at the beginning of May (6 May), because before winter the farmer did not know the exact location of the crop within the farm yet.

In the flower strips, direct visual sampling was performed once a week during the bloom period to assess the floral coverage in every plant species, based on an adapted scale [57]: 1 (>0–1%), 2 (1–10%), 3 (10–25%), 4 (25–50%), 5 (50–100%) and the number of flowers (*B. officinalis*, *N. gallica*, *S.vulgaris, V.sativa,*) or inflorescences (*C. officinalis*, *C. sativum*, *D. catholica*, *D. virgata*, *L. maritima*, *S. verbenaca*) was also counted. In La Poveda, 3 marked areas (1 × 1 m^2^) were randomly distributed in each of the 2 flower strips of each flower plot (6 in total per plot). Because the flower coverage and season can have an influence on the plant attractiveness and on the activity of the pollinator species, the total sampling period was divided into 3, in order to allow for comparison of the pollinator groups visiting the plant species with simultaneous bloom: (1) early spring blooming flowers; (2) late spring-early summer blooming flowers; (3) summer co-blooming flowers with the crop. In the commercial field of Corral de Almaguer, 4 marked areas (1 × 1 m^2^), ≈70 m apart, were distributed in the flower stip.

### 2.5. Visitor Sampling

Visual samplings of pollinator visits were performed weekly in the flower strips and crop, between 09:00 and 14:00 under suitable weather conditions for foraging visitors (temperature above 16 °C, clear skies and calm wind). Every week we started in a different block in both the experimental and the commercial plots. Depending on the peculiarities of experimental or commercial field and flower strip or crop, the samplings were made differently.

In the experimental plots (La Poveda), the flower visits in the flower strip or to melon crop were assessed. In the flower strips, observations were done in the marked areas previously described (6/plot, 3 min/marked area, 18 min/plot in total). Visits in the melon crop were assessed in transects 10 m long × 1 m wide (3/plot, 3 min/transect, 9 min/plot in total) which yielded more visits. In preliminary samplings with fixed marked areas, the number of melon flowers could be zero in some sampling dates, due to their staggered blooms.

In the commercial field of Corral de Almaguer, the flower emergence in the flower strip was not as homogeneous as in the experimental plots, and pollinators were recorded in transects 15 m long × 1 m wide over 3 min. Four transects 70 m apart, were located in the flower strip and in the melon crop in each of the 7 different distances from the flower strip (Figure 1). To minimize the influence of the number of flowers in the flower strip, the bee visitation rate (visits· flower^−1^) was used to compare visits to the flower strip and crop. Therefore, the number of flowers was also counted in 3 marked areas (1 × 1 m^2^) per transect in the flower strip, and in each of the 28 transects of the melon crop, in order to estimate the number of flowers observed per transect.

The visitor groups considered in the visual samplings were beetles, hoverflies and bees. Lepidopterans and Vespoidea were very scarce and were not included in analysis. Bee species were only identified up to family or genus but for statistical purposes, they were classified following the widely accepted phylogenetic family classification based on the proboscis length [58], which helps in structuring bee communities and plant-pollinator networks as it is related to the to the ability of pollinators to access floral rewards. The specimens in the visual sampling were assigned to the group of long-tongue (L-T) bees (tongues normally longer than 6 mm; Apidae and Megachilidae) or short-tongue (S-T) bees (tongues normally shorter than 5 mm; Colletidae, Halictidae, Melittidae, Andrenidae) [58,59]. The last group included bees with very different body sizes, so finally we considered 3 functional bee groups: small (<1cm) short-tongued (S-T), large S-T and long-tongued (L-T) bees—because body size, alongside proboscis length, matters for the choice of the flower.

To become acquainted with the most common pollinators in our study areas, and to help us understand the species richness in every visitor group of the visual samplings, some destructive samplings were also undertaken. In the first year of the study, bi-weekly captures with an entomological sweep net, or 3 square methacrylate wet pan traps (25 cm side) with the bottom painted in fluorescent yellow (F201 yellow^®^, Paintusa, Valencia, Spain) were performed. Additionally, throughout the years, individual species were captured with an entomological net and taken to the lab for identification. Those visitors captured in the melon flowers were noted down. Species present in Central Spain were identified according to the Atlas of Hymenoptera [60] and Ortiz-Sánchez [61], and some of them were already well-known [32,52,55].

### 2.6. Melon Productivity and Quality 

Even though melon has staggered ripening, a single harvest when the first fruits were fully mature was performed. All fruit from every plot in the experimental farm (La Poveda) were collected (13 August 2013 and 5 August 2014). In the commercial field of Corral de Almaguer, 5 distances from the flower strip (1.75, 10.5, 28, 45.50 and 71.75 m) and 3 randomly located transects, 15 m long per distance, were selected. All mature melons were collected inside 3 frames (1 × 1 m^2^) randomly located per transect (31 July 2014). Collected fruits were weighed in the field to calculate the mean fruit weight and yield per ha (dynamometer ProScale^®^ Versa 77, Fletcher, NC., USA).

A selection of 12 typically sized melons for the variety (2–4 kg) without external defects, randomly selected in every plot at La Poveda, and 9 melons in every distance at Corral de Almaguer were taken to the lab for quality parameter measurements (following Cabello et al. [62]). In the lab, the fruit diameter and length were recorded along with the following parameters: flesh thickness from the placenta to the beginning of the exocarp (caliber Krefting, Haan, Germany); flesh firmness (penetrometer fitted with an 8 mm diameter probe Bertuzzi FT 327^®^, Facchini, Busto Arsizio VA, Italy); percentage pulp juice or juiciness, measuring the pulp fresh (electronic balance FY-3000^®^, A&D, Tokio, Japan) and pulp liquefied weight (blender model 753^®^, Moulinex, Mayenne, France); pH of the juice (pH meter Basic 20^®^, Crison, Spain) and total soluble solids expressed as °BRIX (refractometer Palette 100^®^, Atago, Tokyo, Japan). To evaluate the efficiency of insect pollination, in 2013 we weighed the placenta and seeds, while in 2014 we calculated the total number of seeds per fruit as a more precise indicator [63].

### 2.7. Statistical Analysis

In La Poveda experimental farm, linear mixed-effect models (LMM) [64,65] were used to analyze the pollinator visits. In the flower strips, in order to assess the attractiveness of the different plants species to each visitor group, we considered the mean number of visits in the 6 marked areas per plot (18 min in total) as the dependent variable and visitor group (beetles, hoverflies, L-T bees, small S-T bees and large S-T bees) and plant species as the fixed factors. Plants that did not have a high percentage of coverage (<1%) and/or attracted few pollinators were not considered for statistical analysis (*N. gallica, S. verbenaca*., *S. vulgaris, V. sativa* and *D. catholica*). The block was considered as a random factor and the sampling dates as the repeated measures factor. We included as covariate the number of flowers or inflorescences of each plant species. Separate analyses were carried out in every bloom period—(1) early spring blooming flowers; (2) late spring-early summer blooming flowers; (3) summer co-blooming flowers with the crop—and year, because the plant composition, associated visitors and plot location within the farm changed yearly. In the melon crop, to compare the visits between melon plots with and without flower strips and to ascertain its role as pollinator competitor or facilitator, the mean number of visits in the 3 transects per plot (9 min in total) was the dependent variable; the treatments (control and flower strip plots) and visitor group (L-T bees, small S-T bees and large S-T bees) were the fixed factors; the block was the random factor; and the sampling dates were the repeated measures factor. Facilitation occurred when visits to melon plots with flower strips were significantly higher than to control plots; competition, if the number was significantly lower; and no effect if statistical differences were not observed between treatments. Data in all cases were transformed to [ln (x + 1)] for normality prior analysis. The lowest value of Akaike’s information criterion (AICc) was used to select the best covariance structure for the repeated measures factor [64,65] and the linearly independent pairwise comparisons of estimated marginal means were performed using the Fisher least significant difference (LSD) test (*p* < 0.05).

In the Corral de Almaguer commercial field, a generalized linear mixed model (GzLMM) was performed in R version 3.0.2 (R Core Team, Vienna, Austria) using the glmmADMB package to evaluate whether the bee visitation rate in the melon crop declined with increasing distance from the flower strips. We accounted for overdispersion by fitting a negative binomial error distribution and using a log link function. We used the visits per transect (3 min) as a dependent variable, the number of flowers as an offset and the distance (8 measured distances, range 0–100 m) as a continuous fixed effect. To account for non-independence of data collected we included the block and the sample dates as random factors. We tested the significance of the main effect using likelihood ratio two-sided test, and Tukey-test (*p* < 0.05) to contrast the distant levels.

In La Poveda experimental farm, the melon productivity and quality parameters in control and flower strip plots were compared every year independently using a Student’s *t*-test (α < 0.05). If any of the assumptions was violated after variable transformation [ln (x + 1)], a non-parametric Mann–Whitney/Wilcoxon test (*p* < 0.05) was applied. In Corral de Almaguer commercial field, the melon productivity and quality parameters at different distances from the flower strip were analysed with one-way analysis of variance (ANOVA). Means were separated with the LSD multiple range test (*p* < 0.05). The non-parametric Kruskal–Wallis test (*p* < 0.05) was used to establish differences when data violated the premises of the ANOVA.

## 3. Results

### 3.1. Bloom Period and Flower Coverage in the Flower Strips

**Figure 2:**
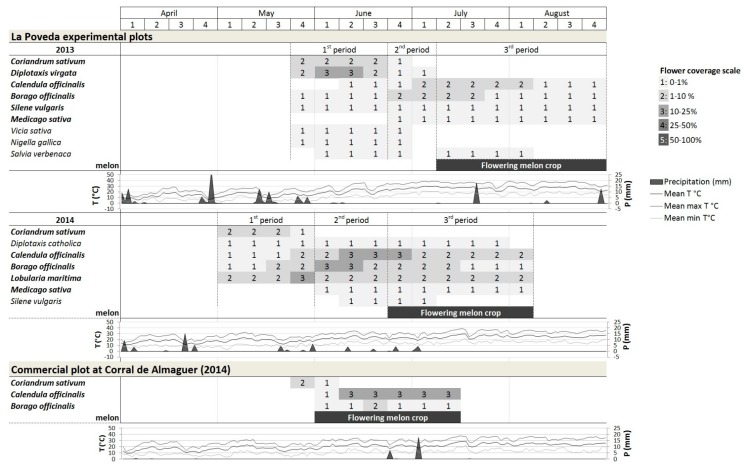
Floral coverage in the flower strips of La Poveda experimental plots (Madrid) during the three bloom periods and of the commercial melon field at Corral de Almaguer (Toledo) and meteorological conditions (temperature and precipitation [53]), The plants in bold were used in the statistical analyses of comparison between visitor groups and plant species each year.

The floral coverage, together with the precipitation and the daily mean, maximum-mean and minimum-mean temperatures during the sampling period in both farms, is presented in Figure 2.

#### 3.1.1. La Poveda Experimental Plots

The year 2013 was rainier (145.9 mm) than 2014 (65.4 mm), and milder (mean 20.2 ± 3.0 °C; maximum mean 34.0 ± 2.4 °C) than 2014 (mean 21.4 ± 1.9 °C; maximum mean 29.1 ± 2.0 °C), which also had a more delayed spring (minimum mean up to mid May 5.9 ± 2.6 °C). 

The bloom sampling period of the flower strip lasted 13 weeks both years. In the first bloom period, in terms of floral coverage, *D. virgata* and *C. sativum* were predominant in 2013 and *C. sativum* and *L. maritima* in 2014. Due to an error by the seed supply company, in 2014 a different species of *Diplotaxis* emerged: *D. catholica* L., with a longer bloom, yet a height and coverage considerably lower than that of *D. virgata*. Over the two years, the most flower rich species in the second and third bloom periods were *C. officinalis* and *B. officinalis*. In the second period of 2013, there was competition for space among some plant species: *D. virgata* produced a huge amount of dried matter, and the floral coverage of *C. officinalis* and *B. officinalis* was much lower than in 2014. *Medicago sativa* reached the maximum bloom in the third period, coinciding with the melon bloom. The plant species with the highest floral coverage in the flower strip had open nectaries, except *B. officinalis* and *M. sativa,* which had partly concealed or concealed nectaries, respectively. The rest of the species contributed less to the floral coverage (Figure 2).

#### 3.1.2. Commercial Field at Corral de Almaguer

In 2014, it was drier in this area compared to La Poveda (25.87 mm of rain). The mean temperature was 20.09 ± 0.36 °C; the maximum mean temperature 28.49 ± 0.43 °C, and the minimum mean temperature 11.09 ± 0.32 °C. 

The bloom period of the flower strip was recorded over seven weeks. Only *C. sativum*, *C. officinalis* and *B. officinalis* reached coverage percentages >1%. The flowering of *C. sativum* (mid-May to mid-June) was not coincident with melon blossom but those of *C. officinalis* (three in floral coverage; Figure 2) and *B. officinalis* (1–2 in flower coverage; Figure 2) were.

### 3.2. Visitors

During the bloom period of the flower strips and crop, the destructive sampling with sweep nets and pan traps illustrated the great richness of insects visiting the flowers: bees (69 species from 20 genera), beetles (19 species) and hoverflies (nine species) (Table 2). In the bee group, the families Apidae, Megachilidae, Andrenidae and Halictidae were well represented.

#### 3.2.1. La Poveda Experimental Plots

**Figure 3:**
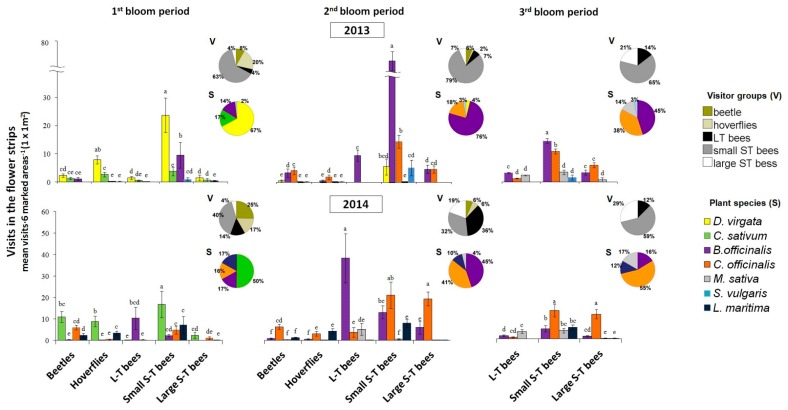
Visits (mean ± standard error (SE)) of beetles, hoverflies, small (<1cm) short-tongued (S-T), large S-T and long-tongued (L-T) bees to different plants of a flower strip in La Poveda experimental plots, in different years and bloom periods. Means are observations of three blocks (six marked areas (1 × 1 m^2^)/plot, 3 min/marked area, 18 min in total) and those followed by the same letter are not significantly different within bloom periods and years. Linear mixed-effects model; Fisher’s least significant difference (LSD) post hoc; *p* < 0.05. Number of flowers of each plant species was included as covariate. The pie-charts show the percentage of the different visitor groups or plant species within bloom periods and years.

In the flower strips, significant differences were found between visitor groups, plant species and their interaction in all blooming periods and years (Table 3). The most visited plants for each visitor group are shown in Figure 3. In every bloom period and year, the different plant species were visited significantly more by certain visitor groups. In the first bloom period of 2013, *D. virgata* was highly attractive to small S-T bees and hoverflies, and *B. officinalis* to small S-T bees. In 2014, however, *D. virgata* was not present in the flowering plant mix and the small S-T bees preferentially visited *C. sativum*. In the second bloom period, *B. officinalis* and *C. officinalis* were the most attractive plant species to small S-T bees in 2013 but in 2014, *B. officinalis* was highly visited by L-T bees, and *C. officinalis* by both small and large S-T bees. In the third bloom period, *B. officinalis* was the most visited plant by small S-T bees in 2013, and in 2014, the highest number of visits of small and large S-T bees was recorded in *C. officinalis* (Figure 3). The number of flowers in the plant species also affected the pollinator visits and, in general, those with the highest number of flowers received more visits (Table 3).

In general, beetles appeared early in the season in our area, were abundant in the first bloom period of the flower strip in La Poveda in 2014 (25% of visits), and their populations lowered in the second period (6%). They practically disappeared in the third period, which coincided with the melon bloom. Their lowest abundance was recorded in 2013 (8%), the year with the longest and coolest winter and the latest flower strip bloom. Hoverflies followed a similar pattern to that of beetles, but their abundance was generally much lower. The L-T bees and the large S-T bees were especially abundant in the second and third bloom periods. In general, the small S-T bee group had the highest number of visits in flowering plant strips in all bloom period and years (32–79%) (Figure 3).

**Figure 4:**
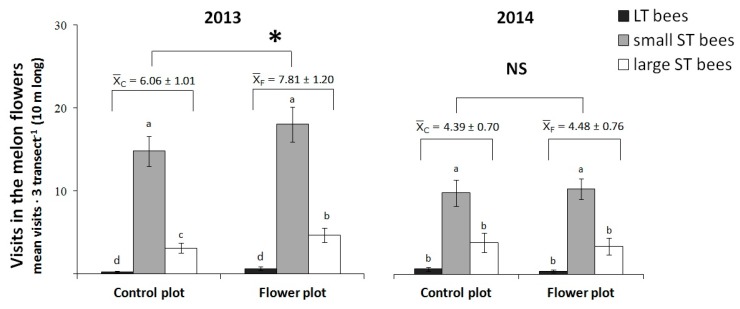
Visits (mean ± SE) to melon flowers in control plots or plots with flower strip of La Poveda experimental farm. * Indicates significant differences between treatments. NS = non-significant difference. Bee groups: small (<1 cm) short-tongued (S-T); large S-T and long-tongued (L-T) bees. Means are observations of three blocks (three transects/plot, 3 min/transect, 9 min in total) and those followed by the same letter are not significantly different within years. Linear mixed-effects model, Fisher’s LSD post hoc, *p* < 0.05.

In the melon crop, bee species (L-T, small S-T, large S-T bees) were the only visitors of melon flowers (Figure 4), and significant differences were detected among visitor groups both years (2013: F_287.6_ = 232.8, *p* = < 0,001; 2014: F_212.1_ = 17.4, *p* = < 0,001; Figure 4). Only in 2013 did the flower strip act as facilitator and the total number of visits was significantly higher in the melon plots with flower strips (2013: F_187.6_ = 5.70, *p* = 0.019; 2014: F_112.1_ = 0.05, *p* = 0.823; Figure 4). The interaction visitor group- treatment was not significant in either year (2013: F_287.6_ = 0.44, *p* = 0.664; 2014: F_212.1_ = 0.17, *p* = 0.849). The visits of small S-T bees were the most abundant in both melon control plots and melon with flower strip plots. The identified visitors in the melon flowers from the destructive sampling with the sweep net are shown in bold in Table 2.

#### 3.2.2. Commercial Field at Corral de Almaguer

**Figure 5:**
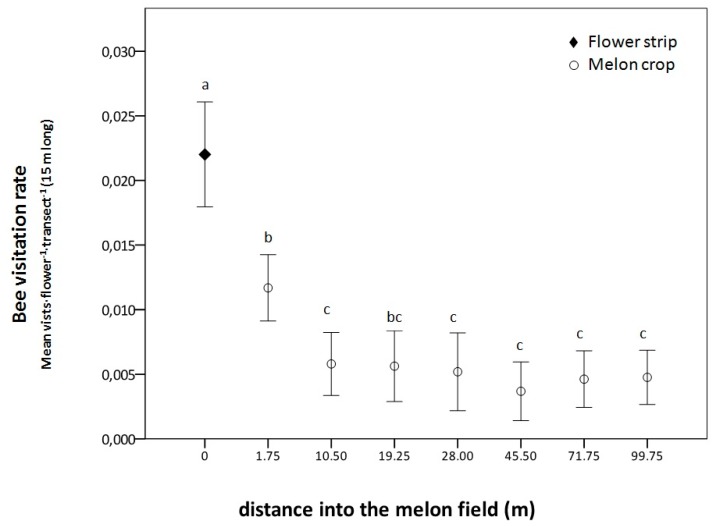
Bee visitation rate (mean visits·flower^−1^ ± SE) in the flower strip and at different distances of the melon crop in the commercial field of Corral de Almaguer (Central Spain). Means are observations per flower and transect 15 m in length during 3 min, and those followed by the same letter within each distance are not significantly different. Generalized linear mixed model; Tukey post hoc tests; *p* < 0.05.

Bee visitation rate in the flower strip was significantly higher than in the melon flowers, where they were significantly affected by the distance to the flower strip (χ^2^_(__df)_ = 1117.5_(7),_ *p* < 0.001, Figure 5). Visitation rate to the melon flowers decreased with an increase in distance to the flower strip and were significantly higher at the first distance (1.75 m) compared to other distances (10.5 to 100 from the flower strip), except for the third (19.25 m).

### 3.3. Melon Quality and Production

In general, both in the small experimental plots at La Poveda and in the commercial field at Corral de Almaguer, we did not find any statistically significant difference in the production (fruit yield in tons per ha) or in the quality parameters of the melon fruits (fruit weight in Kg; fruit diameter and fruit length in cm; flesh thickness in cm; flesh firmness in Newtons; % juiciness; ^o^ BRIX; pH; placenta plus seed weight in g and/or number of seeds) in the years of the study between control melon plots and plots with flower strips or between distance to the flower strip. In 2014, in the experimental plots at La Poveda, control melon plots yielded more fruit tons hA^−^^1^ (31.0 ± 0.2) than the flower plots (24.4 ± 0.1) (*t* = −2.43, *p* = 0.024) with a significantly higher fruit weight (kg) (control plots: 2.3 ± 0.1; flower plots 2.1 ± 0.1) (*W* = 1482.5, *p* = 0.015). In 2013 in La Poveda, and in 2014 in the commercial field of Corral de Almaguer, no statistically significant difference was detected.

## 4. Discussion

### 4.1. Selection of Suitable Flowering Plants

Flowering plant strips contribute foraging habitats to many pollinators by offering food, shelter and nesting resources. The increased plant diversity and the availability of flowers throughout the season (e.g., plants with staggered and/or precocious bloom) contributes to the enhancement of bee populations over time [27,36,66,67,68,69]. In our experimental plots, the sequential bloom lasted for 13–14 weeks and allowed the bee, hoverfly and beetle species to visit the flower strips and to use their nectar and pollen resources during two seasons (spring and/or summer). The beginning of the bloom in the flower strips was earlier than usual in the second year (the mean and the maximum mean temperatures increased during the years of the study) and this affected the visitor groups associated with the different bloom periods.

The most suitable species in terms of coverage and attractiveness were *C. sativum* and *D. virgata* in spring, and *B. officinalis* and *C. officinalis* in summer. All these species are well known for their attractiveness to pollinators when sown as mono-specific plots [29,30,32,70] and they also behaved very well in our mix. All these plants had the highest floral coverage, and they were the tallest in the mixture (between 50–100 cm in our case), which probably facilitated the ability of pollinators to find them. They have open or partly concealed nectaries and were visited mostly by S-T bees—also predominant in the melon crop—because the presence of specialised feeding structures in the pollinators (e.g., long proboscis) are not required to obtain a flower reward. However, *B. officinalis* was also visited by a wide range of L-T bees but only before the melon bloom. Some L-T bees are considered more efficient pollinators of *B.officinalis* than the S-T Halictidae species, which did not touch the flower stigma while drinking nectar [71].

On the contrary, at both locations (La Poveda and Corral de Almaguer), other species were not considered good candidates for the mixture and were not sown the following years for several reasons. Pollinators were not attracted to these plants most likely because they did not exhibit some of the required features for attraction (e.g., high floral coverage and/or good plant height) or because the blooming period was short. The species *N. gallica*, *S. verbenaca*, *V. sativa*, *S. vulgaris, D. catholica* and *M. sativa* had little contribution to the total coverage due to their low bloom and received fewer visits than the most attractive plants. All these plants—except *N. gallica*—have concealed nectaries, which means extra work to gain the reward compared to the most attractive plants in our study *(C. sativum, D. virgata, B. officinalis, C. officinalis*), which have open or partially concealed nectaries. Moreover, the short bloom duration and low height of *N. gallica* and *V. sativa,* also most likely contributed to the low number of visits recorded. In La Poveda, the species *L. maritima,* was sown for the first time in 2014 because it is highly attractive to hoverflies [72,73]. This species emerged well and exhibited a high percentage of floral coverage but, in agreement with Barbir et al. [32], received almost no visits within the mixture. Its low height (<20 cm) seems to have accounted for this, because taller plants hide the visual flower signals of color, which are particularly important for pollinator recognition [74].

Other factors to take into account for the success of a given species within a mixture are the speed of senescence, and the final dried mass reached. The species *D. virgata* ended its development and dried in early summer (end of June–mid July), reaching a large mass that occupied the space needed for the regular growing of the nearby species. In 2014, when the *Diplotaxis* species of lower height was present by error in La Poveda (*D. catholica*), the coverage percentage of *B. officinalis* and *C. officinalis* was higher, probably because they had more space for growing and were not shaded by the taller *D. virgata*. By contrast, the dried mass of other plant species reached a much lower volume.

### 4.2. Visits to the Melon Crop and the Role of the Flower Strip as Competitor or Facilitator

In the experimental plots of La Poveda, visits to melon flowers were significantly higher in the melon plots with flower strip only in the year 2013, suggesting that the flower strip was acting as an exporter. In 2014, no statistically significant differences were found between the two types of plots, suggesting that its role was neutral for pollinators. The total flower coverage and attractiveness of the blooming species in the flower strip may have accounted for this. Floral coverage in the flower strips was higher in 2014 during the third bloom period compared to 2013, the year in which significant differences were found—probably because the resources in the flower strips were not enough to satisfy the pollinator needs. On the contrary, if resources are high in the flower strips, pollinators would probably not be interested in searching for food in the melon crop due to its less attractive flowers [50] and, therefore, the flower strip would not act as a facilitator. The number of visits to the flower strip was similar in 2013 and 2014; however, differences in the visits to melon flowers between the flower and control plots were only observed in 2013. The attractiveness of *C. officinalis* and *B. officinalis* to the small S-T bee group might have accounted for this. We have focused on this group because it was the most abundant in both the flower strip and the melon flowers, and because some small bees belonging to the family Halictidae (genus *Lassioglossum* spp.) have been previously identified as a key pollinators of the melon crop in Central Spain, such as the eusocial *L. malachurum* (Kirby, 1802) [52], which is also seen in our study. In 2013, the most visited plant for these bees was *B. officinalis,* which does not have a high pollen content and mainly supplies nectar [75]; therefore, this could have probably generated an increase in pollinator foraging activity, trying to seek pollen in nearby resources such as the melon crop. The eusocial *Lassioglossum malachurum* (Kirby, 1802) is a major pollen-forager species [52,76]. By contrast, according to Hicks et al. [77], *C. officinalis,* the most visited plant in 2014, produces a lot of both pollen and nectar compared to the other 65 plant species, thus preventing the displacement of bees to the melon crop. In Mediterranean landscapes, it is known that Compositae was the most exploited family for the species *L. malachurum* [78]. Besides, melon has a low number of open flowers each day, the flowers are relatively hidden and unattractive to pollinators compared to wildflowers, and only offer a small amount of nectar and pollen [50]. The results highlight the fact that both resource quantity and quality matter to flower visitors [79], because pollinators are able to distinguish between plant species and learn which ones provide the greatest reward [80].

In the commercial field of Corral de Almaguer, the flower strip was, as expected, much more attractive to bees than the nearby melon flowers, and this agrees with the results of other studies [22,36,47,81]. The landscape context seems to be important in determining the density of some pollinators in the flower strips, e.g., bumblebees [82], which were not very common in our commercial farm. In agreement with Kohler et al. [37], we also found that the effect of our flower-rich strip was spatially limited. Visits in the first distances in the melon crop (<2 m) were higher compared to the farthest distances. The decline of pollinator visits to the crop with the increase in distance to natural of semi-natural areas has also been reported [37,83,84]. Our finding supports the results of La Poveda in 2014, because the plant with the highest floral coverage in the flower strip of the commercial field was *C. officinalis*, which seemed not to act as an exporter of pollinators beyond 2 m.

### 4.3. Melon Productivity and Quality

The significant differences recorded on the bee visits between flower and control plots in 2013 could have had an influence on the productivity and quality of the melon fruits, as has been shown for other crops [38,41,42,46]. Nevertheless, the presence of flower strips in our small and commercial plots was not associated with an improvement in melon production or quality. In agreement with our findings, the yield of tomato and pepper [45], cider orchards [43], cucumber [44] or commercial strawberry [47] was not affected either. Only in the experimental farm in 2014, the yield and weight of melons from the control plots was higher, in spite of the fact that the number of visits were equal between the two kinds of plots. Therefore, this difference seems to be unrelated to flower visitors but to other factors, such as crop management practices, soil quality, etc. The results in the commercial farm could have also be affected by the presence of *A. mellifera* hives, which could have been enough for an optimal melon pollination. Hence, the flower strip would not have offered an extra advantage. However, wild bees can improve pollination services, in spite of the presence of *A. mellifera* hives [10], and this could have happened in our farm.

In some studies, effects have been detected in the years following to the strip establishment [42]. Probably because wild bee populations need time to colonize new habitats [85], permanent flowering strips in crop fields when possible would also enhance the presence of pollinators in the area over time. However, this initiative seems to be a challenge in annual crops (e.g., melon) in intensive agroecosystems with a rotation period between years [44], because the distance between the flower strip and crop within the farm can exceed its possible area of influence, especially in small bees species, which usually forage within an area of few hundred meters from the nest [86]. Furthermore, pollination services in melon fields could be enhanced by including soil patches, alone or in combination with flowers, with adequate features for *Lasioglossum* females to build nests (e.g., compact soil almost void of vegetation [77]), because species of this genus are key pollinators of the crop.

## 5. Conclusions

Our study provides a list of S-T and L-T pollinators that visit melon fields in Central Spain and identifies some good plant species of high floral coverage and staggered bloom, to be included in flower strips: *C. sativum*, *D. virgata*, *C. officinalis* and *B. officinalis*. Based on our results, the plant composition in the mixture must be carefully chosen. Even though the present study was not designed to evaluate interspecific competition between flower strip plant species, the shorter plant *L. maritima* remained hidden under the highest plants and received a low number of visits, even though its floral coverage was high. Additionally, *D. virgata* produced a large amount of dry matter, which could have diminished the floral coverage of the nearby species. Moreover, in choosing the optimal mix, it is also essential to take into account which species support the key crop pollinator taxa, and to facilitate their movement from the flower strip to the crop. In our area, we suggest that the concurrence of blooming in the rewarding *C. officinalis* with the melon crop should be avoided; otherwise the flower strip may not act as a pollinator exporter of the main pollinator taxa to the melon crop. However, further long-term studies with mono-specific flower strips are needed to confirm our hypothesis.

In our area, the presence of the specific flower strips evaluated in experimental and commercial melon farms did not have an influence on melon productivity and quality. However, offering nesting structures and flowering plants on a regional scale might increase bee pollinator populations and so help to provide adequate pollinator services over the years.

## Figures and Tables

**Table 1:** Characteristics of the specific taxa that make up the flower strips.

Species	Family	Floral Nectaries Location	Height (cm) ^1^	La Poveda	Corral de Almaguer
*Calendula officinalis* L.	Compositae	open	20–50	2013, 2014	2014
*Coriandrum sativum* L.	Umbelliferae	open	40–60	2013, 2014	2014
*Nigella gallica* L.	Ranunculaceae	open	10–40	2013	2014
*Lobularia marítima* L.	Cruciferae	open	2–20	2014	-
*Borago officinalis* L.	Boraginaceae	partly concealed	30–70	2013, 2014	2014
*Diplotaxis virgata* Cav.	Cruciferae	partly concealed	50–100	2013	-
*Diplotaxis catholica* L.	Cruciferae	partly concealed	<80	2014	2014
*Medicago sativa* L.	Leguminosae	concealed	30–70	2013, 2014	2014
*Salvia verbenaca* L.	Labiatae	concealed	5–60	2013	2014
*Silene vulgaris* Moench.	Caryophyllaceae	concealed	24–80	2013, 2014	2014
*Vicia sativa* L.	Leguminosae	concealed	<80	2013	2014

^1^ Data from Flora Ibérica website [56].

**Table 2:** Visitor species identified after destructive sampling (sweep net and pan traps) in experimental and commercial melon fields of Central Spain.

**Beetles**			
**Bruchidae**	***Spermophagus*** **sp.**	**Curculionidae**	**Apion sp.1**
	*Anthaxia anatolica* Chevrolat 1837 ^1^	Dasytidae	*Enicopus calcaratus* Kiesenwetter 1859 ^1^
Cerambycidae	*Agapanthia cardui* (Linnaeus 1767) ^1^		*Psilothrix viridicoerulea* (Geoffroy 1785) ^1^
	*Agapanthia annularis* (Olivier 1795) ^1^	Dermestidae	*Attagenus fasciatus* (Thunberg 1795) ^1^
	*Certallum ebulinum* (Linnaeus 1767) ^1^	Meloidae	*Cerocoma schaefferi* (Linnaeus 1758) ^1^
Cetoniidae	*Oxythyrea funesta* (Poda 1761) ^1^	Nitidulidae	*Meligetes* sp^.1^
	*Tropinota hirta* (Poda 1761) ^1^	Oedemeridae	*Oedemera podagrariae* (Linnaeus 1767) ^1^
Chrysomelidae	*Altica* sp. ^1^		*Oedemera simplex* (Linnaeus 1767) ^1^
	*Clytra* sp. ^1^	Tenebrionidae	*Heliotaurus ruficollis* (Fabricius 1781) ^1,2^
Coccinellidae	*Coccinella septempunctata* Linnaeus 1758 ^1,2^		
**Hoverflies**			
Syrphidae	*Ceriana vespiformis* (Latreille 1804) ^1^		*Scaeva* sp.^1,2^
	*Episyrphus balteatus* (De Geer, 1776) ^1,2^		*Sphaerophoria rueppellii* (Weidemann, 1820) ^1^
	*Eristalis tenax* (Linnaeus, 1758) ^1,2^		*Sphaerophoria scripta* (Linnaeus, 1758) ^1,2^
	*Eupeodes corollae* (Fabricius, 1794) ^2^		
**Long-Tongue (L-T) bees**			
Apidae	*Amegilla quadrifasciata* (de Villers, 1789) ^1,2^		*Eucera notata* Lepeletier, 1841 ^1^
	*Anthophora agama* Radoszkowski, 1869 ^1^		*Habropoda zonatula* Smith, 1854 ^1^
	*Anthophora atroalba* Lepeletier, 1841 ^1^		*Xylocopa violacea* (Linnaeus, 1758) ^1,2^
	*Anthophora fulvitarsis* Brullé, 1832 ^1^	Megachilidae	***Anthidium florentinum*** **(Fabricius, 1775)** ^1^
	***Apis mellifera*** **Linnaeus, 1758** ^1,2^		*Coelioxys echinata* Förster, 1853 *^,1^
	*Bombus terrestris* (Linnaeus, 1758) ^1,2^		*Hoplitis antigae* (Pérez, 1895) * ^1^
	***Ceratina chalcites*** Germar, 1839 *^,1^		*Hoplitis* sp.*^,1^
	***Ceratina cucurbitina*** (Rossi, 1792) * ^,1,2^		*Megachile pilidens* Alfken, 1924 ^1,2^
	*Ceratina nigrolabiata* Friese, 1896 ^1^		*Megachile rotundata* (Fabricius 1787) ^1^
	*Eucera elongatula* Vachal, 1907 ^1,2^		*Megachile versicolor* Smith, 1844 ^1^
**Small Short-Tongue (S-T) bees**			
Andrenidae	***Andrena bicolor*** **Fabricius, 1775 ^2^**		***Halictus maculatus*** **Smith, 1848** ^1^
	*Andrena djelfensis* Pérez, 1895 ^1,2^		*Halictus seladonius* (Fabricius, 1794) ^1^
	*Andrena tenuistriata* Pérez, 1895 ^1^		*Halictus tetrazonius* Klug in Germar, 1817 ^1^
	*Andrena* sp.1^1^		*Halictus* sp. 1 ^1^
	*Andrena* sp.2 ^1^		*Halictus* sp. 2 ^1^
	*Panurgus calcaratus* (Scopoli, 1763) ^1^		*Lasioglossum discum* (Smith 1853) ^1^
	*Panurgus canescens* Latreille, 1811^1^		*Lasioglossum leucozonium* (Schrank, 1781) ^1,2^
	*Panurgus* sp. ^1^		*Lasioglossum clypeare* (Schenck 1853) ^1^
Halictidae	*Ceylalictus variegatus* (Fabricius, 1798) ^1^		***Lasioglossum malachurum*** **(Kirby, 1802) ^1,2^**
	*Halictus crenicornis* Blüthgen 1923 ^1^		***Lasioglossum minutulum*** **(Schenck 1853) ^1,2^**
	*Halictus gemmeus* Dours, 1872 ^1^		*Sphecodes croaticus* Meyer 1922 ^1^
**Large Short-Tongue (S-T) bees**			
Andrenidae	*Andrena albopunctata ssp. melona* Warncke, 1967 ^1,2^		*Halictus quadricinctus* (Fabricius, 1776) ^1,2^
	*Andrena bicolorata* (Rossi, 1790) ^1^		***Halictus rubicundus*** **(Christ, 1791)** ^1^
	*Andrena bimaculata* (Kirby, 1802) ^1^		***Halictus scabiosae*** **(Rossi, 1790)** ^1,2^
	*Andrena carbonaria* (Linnaeus, 1767) ^1,2^		*Halictus tridivisus* Blüthgen, 1924 ^1^
	***Andrena flavipes*** **Panzer, 1799** ^1^		*Lasioglossum aegyptiellum* (Strand, 1909) ^1^
	*Andrena florea* Fabricius, 1793 ^1^		***Lasioglossum albocinctum*** **(Lucas 1846)**
	*Andrena nigroaenea* (Kirby, 1802) ^1^		***Lasioglossum pygmaeum*** **(Schenck, 1853)** ^1,2^
	*Andrena ovatula* (Kirby, 1802) ^1^		*Pseudapis bispinosa* (Brullé, 1832) ^1,2^
	*Andrena thoracica* (Fabricius, 1775) ^1,2^		*Pseudapis diversipes* (Latreille 1806) ^1^
	*Panurgus banksianus* (Kirby, 1802) ^1^		*Sphecodes albilabris* (Fabricius, 1793) ^1^
	*Halictus asperulus* Pérez, 1895 ^1^		*Sphecodes gibbus* (Linnaeus, 1758) ^1^
Halictidae	*Halictus consobrinus* (Perez, 1895)		*Sphecodes gibbus* (Linnaeus, 1758) ^1^
	*Halictus crenicornis* Blüthgen, 1923 ^1,2^	Melittidae	*Dasypoda visnaga* (Rossi, 1790) ^2^
	***Halictus fulvipes*** **(Klug, 1817)** ^1^		

Visitors were classified in five groups [beetles, hoverflies, L-T bees (Apidae and Megachilidae species), small S-T bees (Andrenidae and Halictidae species ≤ 1 cm) and large S-T bees (Andrenidae, Halictidae and Mellitidae species > 1 cm)]. Bees were categorized according to the size and length of the proboscides [58]: S-T = short-tongue and L-T = long-tongue bees. * Species considered for statistical analysis within S-T bees prior to identification, due to their small size. ^1^ Species present in La Poveda, Madrid; ^2^ Species present in Corral de Almaguer, Toledo. Melon visitors captured with the sweep net are in bold. The taxonomic species name follows Atlas Hymenoptera [60] and Ortiz-Sánchez [61].

**Table 3:** Influence of visitor groups and plant species on the number of visits to the flower strips of La Poveda experimental plots in the different years and bloom periods.

Year	1st Bloom Period			2nd Bloom Period			3rd Bloom Period		
**2013**	**df**	**F**	***p***	**df**	**F**	***p***	**df**	**F**	***p***
Visitor groups (V)	471.2	32.15	<0.001	439.1	79.88	<0.001	222.9	68.46	<0.001
Plant species (S)	3101.5	5.70	0.001	339.9	35.89	<0.001	326.2	141.33	<0.001
V × S	1271.9	19.55	<0.001	1239.0	7.26	<0.001	622.9	20.94	<0.001
N flowers	1141.8	37.01	<0.001	141.5	10.18	0.002	190.5	70.38	<0.001
**2014**									
Visitor groups (V)	448.4	3.95	0.007	437.4	18.17	<0.001	259.8	39.21	<0.001
Plant species (S)	372.8	4.03	0.010	346.8	57.99	<0.001	364.3	24.46	<0.001
V × S	1248.3	3.06	0.003	1237.3	18.48	<0.001	659.8	10.47	<0.001
N flowers	1135.9	5.24	0.024	1110.0	24.02	<0.001	196.8	17.99	<0.001

Visitor groups: beetles, hoverflies, small (<1 cm) short-tongued (S-T); large S-T and long-tongued (L-T) bees. Linear mixed-effects model; *p* < 0.05. Number of flowers of each plant species included as covariate.

## References

[1] Klein A.M., Vaissiere B.E., Cane J.H., Steffan-Dewenter I., Cunningham S.A., Kremen C., Tscharntke T. (2007). Importance of pollinators in changing landscapes for world crops. Proc. R. Soc. B Biol. Sci..

[2] Fontaine C., Dajoz I., Meriguet J., Loreau M. (2006). Functional diversity of plant—Pollinator interaction webs enhances the persistence of plant communities. PLoS Biol..

[3] Garibaldi L.A., Aizen M.A., Klein A.M., Cunningham S.A., Harder L.D. (2011). Global growth and stability of agricultural yield decrease with pollinator dependence. Proc. Natl. Acad. Sci. USA.

[4] Cox-Foster D.L., Conlan S., Holmes E.C., Palacios G., Evans J.D., Moran N.A., Quan P.L., Briese T., Hornig M., Geiser D.M. (2007). A metagenomic survey of microbes in honey bee colony collapse disorder. Science.

[5] Potts S.G., Biesmeijer J.C., Kremen C., Neumann P., Schweiger O., Kunin W.E. (2010). Global pollinator declines: Trends, impacts and drivers. Trends Ecol. Evol..

[6] Van Engelsdorp D., Evans J.D., Saegerman C., Mullin C., Haubruge E., Nguyen B.K., Frazier M., Frazier J., Cox-Foster D., Chen Y. (2009). Colony collapse disorder: A descriptive study. PLoS ONE.

[7] Aizen M.A., Garibaldi L.A., Cunningham S.A., Klein A.M. (2008). Long-Term Global Trends in Crop Yield and Production Reveal No Current Pollination Shortage but Increasing Pollinator Dependency. Curr. Biol..

[8] Breeze T.D., Vaissière B.E., Bommarco R., Petanidou T., Seraphides N., Kozák L., Scheper J., Biesmeijer J.C., Kleijn D., Gyldenkærne S. (2014). Agricultural Policies Exacerbate Honeybee Pollination Service Supply-Demand Mismatches Across Europe. PLoS ONE.

[9] Aizen M.A., Harder L.D. (2009). The Global Stock of Domesticated Honey Bees Is Growing Slower Than Agricultural Demand for Pollination. Curr. Biol..

[10] Garibaldi L.A., Steffan-Dewenter I., Winfree R., Aizen M.A., Bommarco R., Cunningham S.A., Kremen C., Carvalheiro L.G., Harder L.D., Afik O. (2013). Wild Pollinators Enhance Fruit Set of Crops Regardless of Honey Bee Abundance. Science.

[11] Goulson D., Nicholls E., Botías C., Rotheray E.L. (2015). Bee declines driven by combined stress from parasites, pesticides, and lack of flowers. Science.

[12] Kremen C., Williams N.M., Thorp R.W. (2002). Crop pollination from native bees at risk from agricultural intensification. Proc. Natl. Acad. Sci. USA.

[13] Winfree R., Aguilar R., Vázquez D.P., LeBuhn G., Aizen M.A. (2009). A meta-analysis of bees’ responses to anthropogenic disturbance. Ecology.

[14] Cusser S., Neff J.L., Jha S. (2016). Natural land cover drives pollinator abundance and richness, leading to reductions in pollen limitation in cotton agroecosystems. Agric. Ecosyst. Environ..

[15] Garibaldi L.A., Carvalheiro L.G., Leonhardt S.D., Aizen M.A., Blaauw B.R., Isaacs R., Kuhlmann M., Kleijn D., Klein A.M., Kremen C. (2014). From research to action: Enhancing crop yield through wild pollinators. Front. Ecol. Environ..

[16] EC Biodiversity Strategy—Environment—European Commission. https://ec.europa.eu/environment/nature/biodiversity/strategy/index_en.htm.

[17] EC European Commission, Agriculture and Rural Development Greening. https://ec.europa.eu/agriculture/direct-support/greening_en.

[18] Wratten S.D., Gillespie M., Decourtye A., Mader E., Desneux N. (2012). Pollinator habitat enhancement: Benefits to other ecosystem services. Agric. Ecosyst. Environ..

[19] BOE Agencia Estatal Boletín Oficial del Estado Real Decreto 1378/2018. https://www.boe.es/diario_boe/txt.php?id=BOE-A-2018-15349.

[20] Morrison J., Izquierdo J., Hernández E., González-andújar J.L. (2017). Agriculture, Ecosystems and Environment The role of fi eld margins in supporting wild bees in Mediterranean cereal agroecosystems: Which biotic and abiotic factors are important?. Agric. Ecosyst. Environ..

[21] Carvell C., Bourke A.F.G., Osborne J.L., Heard M.S. (2015). Effects of an agri-environment scheme on bumblebee reproduction at local and landscape scales. Basic Appl. Ecol..

[22] Jönsson A.M., Ekroos J., Dänhardt J., Andersson G.K.S., Olsson O., Smith H.G. (2015). Sown flower strips in southern Sweden increase abundances of wild bees and hoverflies in the wider landscape. Biol. Conserv..

[23] Scheper J., Bommarco R., Holzschuh A., Potts S.G., Riedinger V., Roberts S.P.M., Rundlöf M., Smith H.G., Steffan-Dewenter I., Wickens J.B. (2015). Local and landscape-level floral resources explain effects of wildflower strips on wild bees across four European countries. J. Appl. Ecol..

[24] M’Gonigle L.K., Ponisio L.C., Cutler K., Kremen C. (2015). Habitat restoration promotes pollinator persistence and colonization in intensively managed agriculture. Ecol. Appl..

[25] Tuell J.K., Fiedler A.K., Landis D., Isaacs R. (2008). Visitation by Wild and Managed Bees (Hymenoptera: Apoidea) to Eastern U.S. Native Plants for Use in Conservation Programs. Environ. Entomol..

[26] Isaacs R., Tuell J., Fiedler A., Gardiner M., Landis D. (2009). Maximizing arthropod-mediated ecosystem services in agricultural landscapes: The role of native plants. Front. Ecol. Environ..

[27] Balzan M.V., Bocci G., Moonen A.-C. (2014). Augmenting flower trait diversity in wildflower strips to optimise the conservation of arthropod functional groups for multiple agroecosystem services. J. Insect Conserv..

[28] Campbell A.J., Biesmeijer J.C., Varma V., Wäckers F.L. (2012). Realising multiple ecosystem services based on the response of three beneficial insect groups to floral traits and trait diversity. Basic Appl. Ecol..

[29] Carreck N.L., Williams I.H. (2002). Food for insect pollinators on farmland: Insect visits to flowers of annual seed mixtures. J. Insect Conserv..

[30] Hogg B.N., Bugg R.L., Daane K.M. (2011). Attractiveness of common insectary and harvestable floral resources to beneficial insects. Biol. Control.

[31] Carrié R.J.G., George D.R., Wäckers F.L. (2012). Selection of floral resources to optimise conservation of agriculturally-functional insect groups. J. Insect Conserv..

[32] Barbir J., Badenes-Pérez F.R., Fernández-Quintanilla C., Dorado J. (2015). The attractiveness of flowering herbaceous plants to bees (Hymenoptera: Apoidea) and hoverflies (Diptera: Syrphidae) in agro-ecosystems of Central Spain. Agric. For. Entomol..

[33] Barbir J., Azpiazu C., Badenes-Pérez F.R., Fernández-Quintanilla C., Dorado J. (2016). Functionality of Selected Aromatic Lamiaceae in Attracting Pollinators in Central Spain. J. Econ. Entomol..

[34] M’Gonigle L.K., Williams N.M., Lonsdorf E., Kremen C. (2017). A Tool for Selecting Plants When Restoring Habitat for Pollinators. Conserv. Lett..

[35] Amy C., Noël G., Hatt S., Uyttenbroeck R., Van De Meutter F., Genoud D., Francis F. (2018). Flower strips in wheat intercropping system: Effect on pollinator abundance and diversity in Belgium. Insects.

[36] Haaland C., Naisbit R.E., Bersier L.-F. (2011). Sown wildflower strips for insect conservation: A review. Insect Conserv. Divers..

[37] Kohler F., Verhulst J., van Klink R., Kleijn D. (2008). At what spatial scale do high-quality habitats enhance the diversity of forbs and pollinators in intensively farmed landscapes?. J. Appl. Ecol..

[38] Carvalheiro L.G., Seymour C.L., Nicolson S.W., Veldtman R. (2012). Creating patches of native flowers facilitates crop pollination in large agricultural fields: Mango as a case study. J. Appl. Ecol..

[39] Morandin L.A., Long R.F., Kremen C. (2014). Hedgerows enhance beneficial insects on adjacent tomato fields in an intensive agricultural landscape. Agric. Ecosyst. Environ..

[40] Carvalheiro L.G., Saraiva A.M., Giannini T.C., Gemmill-Herren B. (2016). Establishing Knowledge Management System for Ecological Interactions. The case of crop pollinators. Pollination Services to Agriculture: Sustaining and Enhancing a Key Ecosystem Service.

[41] Balzan M.V., Bocci G., Moonen A.C. (2016). Utilisation of plant functional diversity in wildflower strips for the delivery of multiple agroecosystem services. Entomol. Exp. Appl..

[42] Blaauw B.R., Isaacs R. (2014). Flower plantings increase wild bee abundance and the pollination services provided to a pollination-dependent crop. J. Appl. Ecol..

[43] Campbell A.J., Wilby A., Sutton P., Wäckers F. (2017). Getting more power from your flowers: Multi-functional flower strips enhance pollinators and pest control agents in apple orchards. Insects.

[44] Quinn N.F., Brainard D.C., Szendrei Z. (2017). Floral Strips Attract Beneficial Insects but Do Not Enhance Yield in Cucumber Fields. J. Econ. Entomol..

[45] Winfree R., Williams N.M., Gaines H., Ascher J.S., Kremen C. (2008). Wild bee pollinators provide the majority of crop visitation across land-use gradients in New Jersey and Pennsylvania, USA. J. Appl. Ecol..

[46] Ganser D., Mayr B., Albrecht M., Knop E. (2018). Wildflower strips enhance pollination in adjacent strawberry crops at the small scale. Ecol. Evol..

[47] Hodgkiss D., Brown M.J.F., Fountain M.T. (2019). The effect of within-crop floral resources on pollination, aphid control and fruit quality in commercial strawberry. Agric. Ecosyst. Environ..

[48] Garbach K., Long R.F. (2017). Determinants of field edge habitat restoration on farms in California’s Sacramento Valley. J. Environ. Manag..

[49] MAPA Ministerio de Agricultura Y Pesca, Alimentación y Medio Ambiente Anuario de Estadística 2018. https://www.mapa.gob.es/es/estadistica/temas/publicaciones/anuario-de-estadistica/2018/default.aspx?parte=3&capitulo=07&grupo=6&seccion=21.

[50] Bomfim I., Freitas B., de Aragão F., Walters S., Pessarakli M. (2016). Pollination in cucurbit crops. Handbook of Cucurbits: Growth, Cultural Practices, and Physiology.

[51] Tschoeke P.H., Oliveira E.E., Dalcin M.S., Silveira-Tschoeke M.C.A.C., Santos G.R. (2015). Diversity and flower-visiting rates of bee species as potential pollinators of melon (*Cucumis melo* L.) in the Brazilian Cerrado. Sci. Hortic..

[52] Rodrigo Gómez S., Ornosa C., Selfa J., Guara M., Polidori C. (2016). Small sweat bees (Hymenoptera: Halictidae) as potential major pollinators of melon (*Cucumis melo*) in the Mediterranean. Entomol. Sci..

[53] SIAR Sistema de Información Agroclimática Para el Regadío Ministerio de Agricultura Y Pesca, Alimentación Y Medio. Ambiente. http://eportal.magrama.gob.es/websiar/Inicio.aspx.

[54] Viñuela E., Adan A., Rodríguez J., Hernando S., Dorado J., Fernández-Quintanilla C., Canomanuel G., Fereres A. (2012). Provision of ecological infrastructures to increase pollinators and other beneficial organisms in rainfed crops in Central Spain. IOBC/wrps Bull..

[55] Azpiazu C., Morales I., Adán Á., Medina P., Fereres A. (2017). Identifying a suitable annual floral mixture and its relative attractiveness to pollinators in Central Spain. IOBC-WPRS Bull..

[56] Flora ibérica Flora ibérica Plantas Vasculares de la Península Ibérica e Islas Baleares. http://www.floraiberica.es/.

[57] Braun-Blanquet J., Lalucat Jo J., de Bolòs O. (1979). Fitosociología: Bases para el Estudio de las Comunidades Vegetales. Spanish Edition of Pflanzensoziologie: Grundzüge der Vegetationskunde.

[58] Michener C.D. (2007). The Bees of the World.

[59] Biesmeijer J.C., Roberts S.P.M., Reemer M., Ohlemüller R., Edwards M., Schaffers A.P., Peeters T., Potts S.G., Kleukers R., Thomas C.D. (2006). Parallel declines in pollinators and insect-pollinated plants in Britain and the Netherlands. Science.

[60] Atlas Hymenoptera Atlas Hymenoptera. http://www.atlashymenoptera.net.

[61] Ortiz-Sánchez F.J. (2011). Lista actualizada de las especies de abejas en Espana (Hymenoptera: Apoidea: Apiformes). Boletín la Soc. Entomológica Aragon..

[62] Cabello M.J., Castellanos M.T., Romojaro F., Martínez-Madrid C., Ribas F. (2009). Yield and quality of melon grown under different irrigation and nitrogen rates. Agric. Water Manag..

[63] Walters S.A., Taylor B.H. (2006). Efects of honey bee pollination on pumpkin fruit and seed yield. HortScience.

[64] Littell R.C., Henry P.R., Ammerman C.B., Littell R.C., Henry P.R., Ammerman C.B. (1998). Statistical analysis of repeated measures data using SAS procedures. J. Anim. Sci..

[65] Wang Z., Goonewardene L.A. (2004). The use of MIXED models in the analysis of animal experiments with repeated measures data. Can. J. Anim. Sci..

[66] Nicholls C.I., Altieri M.A. (2013). Plant biodiversity enhances bees and other insect pollinators in agroecosystems. A review. Agron. Sustain. Dev..

[67] Rosa García R., Miñarro M. (2014). Role of floral resources in the conservation of pollinator communities in cider-apple orchards. Agric. Ecosyst. Environ..

[68] Blüthgen N., Klein A.-M. (2011). Functional complementarity and specialisation: The role of biodiversity in plant—Pollinator interactions. Basic Appl. Ecol..

[69] Sanchez J.A., Carrasco A., La Spina M., Pérez-Marcos M., Ortiz-Sánchez F.J. (2020). How Bees Respond Differently to Field Margins of Shrubby and Herbaceous Plants in Intensive Agricultural Crops of the Mediterranean Area. Insects.

[70] Bugg R.L., Colfer R.G., Chaney W.E., Smith H.A., Cannon J. (2008). Flower Flies (Syrphidae) and Other Biological Control Agents for Aphids in Vegetable Crops. UC ANR.

[71] Gorenflo A., Diekötter T., Van Kleunen M., Wolters V., Jauker F. (2017). Contrasting pollination efficiency and effectiveness among flower visitors of Malva sylvestris, Borago officinalis and Onobrychis viciifolia. J. Pollinat. Ecol..

[72] Colley M.R., Luna J.M. (2000). Relative Attractiveness of Potential Beneficial Insectary Plants to Aphidophagous Hoverflies (Diptera: Syrphidae). Environ. Entomol..

[73] Pineda A., Marcos-García M.Á. (2008). Use of selected flowering plants in greenhouses to enhance aphidophagous hoverfly populations (Diptera: Syrphidae). Ann. la Soc. Entomol. Fr (N.S.)..

[74] Gumbert A. (2000). Color choices by bumble bees (*Bombus terrestris*): Innate preferences and generalization after learning. Behav. Ecol. Sociobiol..

[75] Carreck N.L., Williams I.H. (1997). Observations on two commercial flower mixtures as food sources for beneficial insects in the UK. J. Agric. Sci..

[76] Pisanty G., Klein A.M., Mandelik Y. (2014). Do wild bees complement honeybee pollination of confection sunflowers in Israel?. Apidologie.

[77] Hicks D.M., Ouvrard P., Baldock K.C.R., Baude M., Goddard M.A., Kunin W.E., Mitschunas N., Memmott J., Morse H., Nikolitsi M. (2016). Food for pollinators: Quantifying the nectar and pollen resources of urban flower meadows. PLoS ONE.

[78] Polidori C., Rubichi A., Barbieri V., Trombino L., Donegana M. (2010). Floral resources and nesting requirements of the ground-nesting social bee, Lasioglossum malachurum (hymenoptera: Halictidae), in a Mediterranean semiagricultural landscape. Psyche A J. Entomol..

[79] Fowler R.E., Rotheray E.L., Goulson D. (2016). Floral abundance and resource quality influence pollinator choice. Insect Conserv. Divers..

[80] Goulson D. (1999). Foraging Strategies of Insects for Gathering Nectar an Pollen and Implications for Plant Ecology and Evolution. Perspect. Plant Ecol. Evol. Syst..

[81] Scheper J., Holzschuh A., Kuussaari M., Potts S.G., Rundlöf M., Smith H.G., Kleijn D. (2013). Environmental factors driving the effectiveness of European agri-environmental measures in mitigating pollinator loss—A meta-analysis. Ecol. Lett..

[82] Heard M.S., Carvell C., Carreck N.L., Rothery P., Osborne J.L., Bourke A.F.G. (2007). Landscape context not patch size determines bumble-bee density on flower mixtures sown for agri-environment schemes. Biol. Lett..

[83] Carvalheiro L.G., Seymour C.L., Veldtman R., Nicolson S.W. (2010). Pollination services decline with distance from natural habitat even in biodiversity-rich areas. J. Appl. Ecol..

[84] Campbell A.J., Wilby A., Sutton P., Wäckers F.L. (2017). Do sown flower strips boost wild pollinator abundance and pollination services in a spring-flowering crop? A case study from UK cider apple orchards. Agric. Ecosyst. Environ..

[85] Williams N.M., Minckley R.L., Silveira F.A. (2001). Variation in native bees used for detecting community changes. Conserv. Ecol..

[86] Zurbuchen A., Landert L., Klaiber J., Müller A., Hein S., Dorn S. (2010). Maximum foraging ranges in solitary bees: Only few individuals have the capability to cover long foraging distances. Biol. Conserv..

